# Retraction: Effect of miR-195-5p on cardiomyocyte apoptosis in rats with heart failure by regulating TGF-β1/Smad3 signaling pathway

**DOI:** 10.1042/BSR-20200566_RET

**Published:** 2020-08-07

**Keywords:** apoptosis, cardiomyocyte, heart failure rats, miR-195-5p, TGF-β1/Smad3 signaling pathway

This article is being retracted from Bioscience Reports at the request of the Editor in Chief and Editorial Board following receipt of a notification from a reader, alerting the Journal to unusual repeated background features in the Western blots.

The Editorial Office was also contacted by the listed corresponding author, Yan Yang, via an email address that differed to the one included with the submission. Yan Yang confirmed that they were unaware of the submission and publication of the article, and that they did not conduct any part of this research, hence should not have been listed as an author on this paper.

The corresponding author of the paper has been contacted via the original email address in the published paper to provide a response to the Editorial Office concerns surrounding the authorship of the paper but has not responded.

In light of the concerns raised regarding the Western blots and the authorship of the paper, the Editorial Board stands by the decision to retract the paper.

